# Supplementary material for: Hospital Variation in Cancer Treatments and Survival OutComes of Advanced Melanoma Patients: Nationwide Quality Assurance in The Netherlands
Source: Cancers (Basel). 2021 Oct 11;13(20):5077. doi: 10.3390/cancers13205077 (PMC8533953; doi:10.3390/cancers13205077)
Supplement: Supplementary file 1 [file cancers-13-05077-s001.zip › cancers-1347836-supplementary.pdf]

Hospital variation in cancer treatments and survival outcomes of advanced melanoma patients: nation-wide quality assurance in The Netherlands.

van Breeschoten J. ,van den Eertwegh A.J.M., de Wreede L.C., Hilarius D.L., van Zwet E.W., Haanen J.B., Blank C.U., Aarts M.J.B., van den Berkmortel F.W.P.J., de Groot J.W.B., Hospers G.A.P., Kapiteijn E., Piersma D., van Rijn R.S., Stevense-den Boer M.A.M., van der Veldt A.A.M., Vreugdenhil G., Boers-Sonderen M.J., Suijkerbuijk K.P.M., Wouters M.W.J.M.

Table of contents

Supplementary material 2
- Figure S1. Flow of new systemic therapies 2
- Figure S2. Geographical location melanoma centers 2
- Figure S3. Flow Chart 3
- Table S1. Patient and tumor characteristics of patients in each individual center (2013-2017) 4
- Table S2. Number of newly diagnosed advanced melanoma patients per center 6
- Table S3. Multivariable Cox-regression for OS 7

Supplementary material

Figure S1. Flow of new systemic therapies

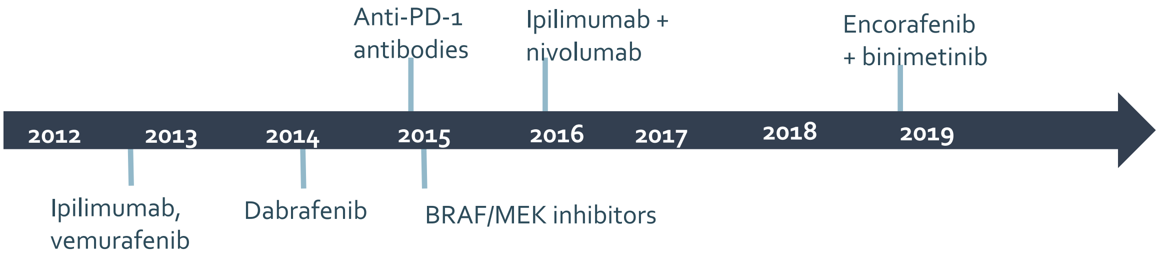

Figure S1. Flow of the introduction of new systemic therapies in the Netherlands in the time period 2012-2019

Figure S2. Geographical location melanoma centers

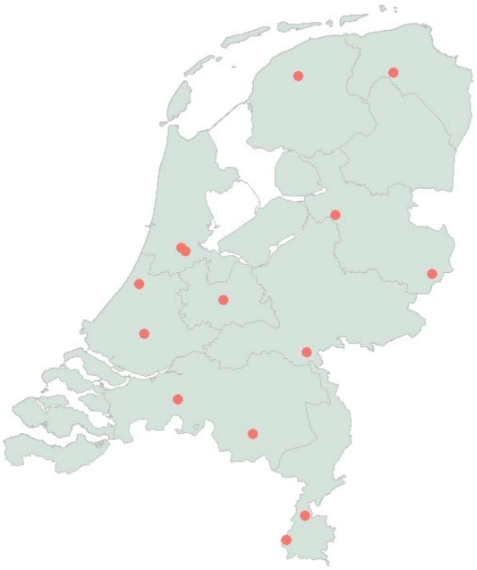

Figure S2. Geographical location of the melanoma centers in the Netherlands. Each center is represented by a red dot.

Figure S3. Flow Chart

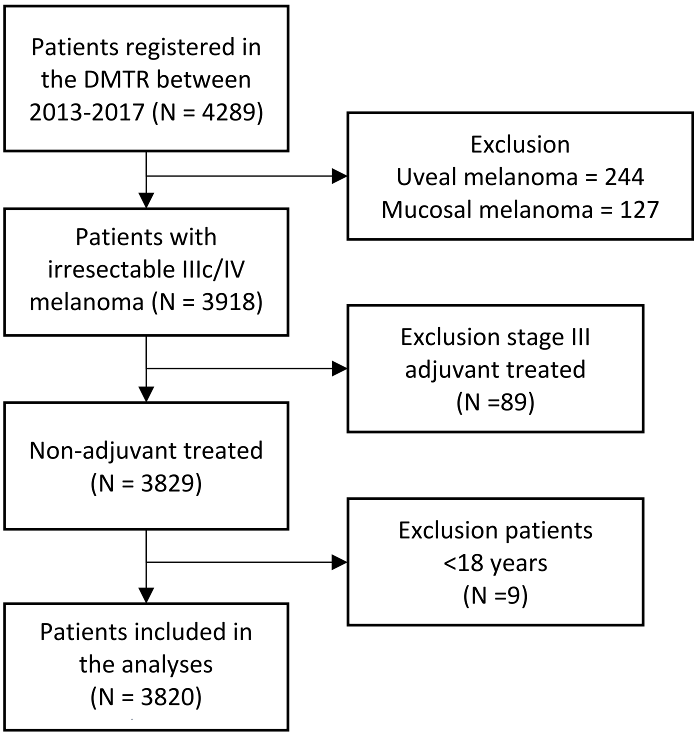

Figure S3. Flow chart of patients included in this study

Table S1. Patient and tumor characteristics of patients in each individual center (2013-2017)

Table S1. *Patient and tumor characteristics of patients in each individual center over the years 2013-2017. Missing data of ≥5% are shown as separate categories.*

**Center ID**		**1**	**2**	**3**	**4**	**5**	**6**	**7**
**N (%)**		353	225	249	205	452	400	174
**Age (median [range])**		64 (21-90)	66 (25-89)	64 (20-94)	65 (30-92)	66 (26-97)	63 (19-89)	65.50 (26-91)
**Gender (%)**	Male	193 (54.7)	143 (63.6)	149 (59.8)	129 (62.9)	275 (60.8)	235 (58.8)	106 (60.9)
	Female	160 (45.3)	82 (36.4)	100 (40.2)	76 (37.1)	177 (39.2)	165 (41.2)	68 (39.1)
**ECOG PS (%)**	0-1	243 (83.5)	184 (82.9)	179 (79.6)	139 (72.8)	335 (85.9)	292 (86.1)	121 (82.3)
	≥2	48 (16.5)	38 (17.1)	46 (20.4)	52 (27.2)	55 (14.1)	47 (13.9)	26 (17.7)
	Unknown	62 (17.6)	3 (1.3)	24 (9.6)	14 (6.8)	62 (13.7)	61 (15.3)	27 (15.5)
**Stage (%)**	IIIc unresectable	22 (6.2)	12 (5.3)	13 (5.3)	4 (2.0)	28 (6.2)	27 (6.8)	6 (3.5)
	IV-M1a	28 (7.9)	9 (4.0)	11 (4.5)	14 (6.8)	32 (7.1)	39 (9.8)	10 (5.8)
	IV-M1b	36 (10.2)	27 (12.0)	23 (9.3)	14 (6.8)	56 (12.5)	51 (12.8)	13 (7.5)
	IV-M1c	267 (75.6)	177 (78.7)	200 (81.0)	173 (84.4)	333 (74.2)	281 (70.6)	144 (83.2)
**LDH (%)**	Normal	208 (60.8)	121 (57.3)	135 (59.5)	109 (57.4)	231 (55.8)	260 (73.2)	96 (57.8)
	1-2x ULN	74 (21.6)	58 (27.5)	61 (26.9)	42 (22.1)	121 (29.2)	50 (14.1)	37 (22.3)
	>2x ULN	60 (17.5)	32 (15.2)	31 (13.7)	39 (20.5)	62 (15.0)	45 (12.7)	33 (19.9)
	Unknown/not determined	11 (3.5)	14 (6.2)	22 (8.8)	15 (7.3)	38 (8.4)	45 (11.3)	8 (4.6)
**Brain metastases (%)**	No	235 (67.1)	167 (74.9)	170 (69.1)	133 (64.9)	332 (73.9)	315 (80.6)	124 (73.4)
	Yes, asymptomatic	32 (9.1)	17 (7.6)	22 (8.9)	16 (7.8)	29 (6.5)	19 (4.9)	12 (7.1)
	Yes, symptomatic	83 (23.7)	39 (17.5)	54 (22.0)	56 (27.3)	88 (19.6)	57 (14.6)	33 (19.5)
**Liver metastases (%)**	No	252 (72.2)	148 (66.4)	165 (66.8)	132 (65.0)	318 (70.5)	279 (71.0)	112 (64.4)
	Yes	97 (27.8)	75 (33.6)	82 (33.2)	71 (35.0)	133 (29.5)	114 (29.0)	62 (35.6)
	Unknown	3 (0.8)	2 (0.9)	3 (1.2)	0 (0.0)	3 (0.7)	9 (2.3)	5 (2.9)
**Organ sites (%)**	<3	190 (53.8)	104 (46.2)	126 (50.6)	98 (47.8)	252 (55.8)	218 (54.5)	98 (56.3)
	≥3	163 (46.2)	121 (53.8)	123 (49.4)	107 (52.2)	200 (44.2)	182 (45.5)	76 (43.7)
**$BRAF^{V600}$ mutation (%)**	Wild-type	153 (43.3)	98 (43.6)	111 (44.6)	90 (43.9)	236 (52.2)	191 (47.8)	66 (37.9)
	Mutant	200 (56.7)	127 (56.4)	138 (55.4)	115 (56.1)	216 (47.8)	209 (52.2)	108 (62.1)

Table S1. *(continued)Patient and tumor characteristics of each individual center of patients diagnosed in 2013-2017. Missing data of ≥5% are shown as separate categories.*

**Center ID**		**8**	**9**	**10**	**11**	**12**	**13**	**14**	**p-value**
**N (%)**		165	191	110	142	87	955	112	
**Age (median (range])**		63 (25-89)	66 (21-90)	67 (21-92)	65 (18-93)	69 (39-90)	60 (23-97)	65.50 (24-88)	<0.001
**Gender (%)**	Male	106 (64.2)	112 (58.6)	65 (59.6)	91 (64.1)	52 (59.8)	533 (55.8)	65 (58.0)	0.343
	Female	59 (35.8)	79 (41.4)	44 (40.4)	51 (35.9)	35 (40.2)	422 (44.2)	47 (42.0)	
**ECOG PS(%)**	0-1	124 (85.5)	143 (92.3)	67 (71.3)	131 (97.0)	50 (78.1)	767 (88.1)	57 (81.4)	<0.001
	≥2	21 (14.5)	12 (7.7)	27 (28.7)	4 (3.0)	14 (21.9)	104 (11.9)	13 (18.6)	
	Unknown	20 (12.1)	36 (18.9)	16 (14.5)	7 (4.9)	23 (26.4)	84 (8.8)	42 (37.5)	
**Stage (%)**	IIIc unresectable	8 (4.9)	13 (7.0)	5 (4.7)	5 (3.6)	5 (5.9)	40 (4.2)	2 (1.9)	0.001
	IV-M1a	13 (8.0)	9 (4.8)	8 (7.5)	9 (6.4)	9 (10.6)	84 (8.9)	22 (20.4)	
	IV-M1b	15 (9.2)	18 (9.7)	6 (5.7)	17 (12.1)	5 (5.9)	104 (11.0)	12 (11.1)	
	IV-M1c	127 (77.9)	146 (78.5)	87 (82.1)	109 (77.9)	66 (77.6)	721 (76.0)	72 (66.7)	
**LDH (%)**	Normal	89 (64.0)	100 (57.5)	60 (61.2)	87 (69.0)	48 (63.2)	598 (66.4)	73 (79.3)	<0.001
	1-2x ULN	31 (22.3)	55 (31.6)	24 (24.5)	23 (18.3)	19 (25.0)	208 (23.1)	13 (14.1)	
	>2x ULN	19 (13.7)	19 (10.9)	14 (14.3)	16 (12.7)	9 (11.8)	94 (10.4)	6 (6.5)	
	Unknown/not determined	26 (15.8)	17 (8.9)	12 (10.9)	16 (11.3)	11 (12.6)	55 (5.8)	20 (17.9)	
**Brain metastases (%)**	No	112 (75.7)	119 (64.3)	67 (67.0)	88 (69.3)	53 (62.4)	679 (71.4)	78 (72.9)	<0.001
	Yes, asymptomatic	5 (3.4)	26 (14.1)	11 (11.0)	7 (5.5)	9 (10.6)	124 (13.0)	5 (4.7)	
	Yes, symptomatic	31 (20.9)	40 (21.6)	22 (22.0)	32 (25.2)	23 (27.1)	148 (15.6)	24 (22.4)	
**Liver metastases (%)**	No	116 (71.2)	134 (72.0)	72 (69.9)	100 (71.9)	54 (62.8)	697 (74.5)	88 (80.7)	0.019
	Yes	47 (28.8)	52 (28.0)	31 (30.1)	39 (28.1)	32 (37.2)	238 (25.5)	21 (19.3)	
	Unknown	2 (1.2)	5 (2.6)	7 (6.4)	3 (2.1)	1 (1.1)	20 (0.2)	3 (2.6)	
**Organ sites (%)**	<3	89 (53.9)	111 (58.1)	49 (44.5)	82 (57.7)	38 (43.7)	525 (55.0)	67 (59.8)	0.053
	≥3	76 (46.1)	80 (41.9)	61 (55.5)	60 (42.3)	49 (56.3)	430 (45.0)	45 (40.2)	
**$BRAF^{V600}$ mutation (%)**	Wild-type	79 (47.9)	89 (46.6)	55 (50.0)	63 (44.4)	40 (46.0)	418 (43.8)	52 (46.4)	0.200
	Mutant	86 (52.1)	102 (53.4)	55 (50.0)	79 (55.6)	47 (54.0)	537 (56.2)	60 (53.6)	

Table S2. Number of newly diagnosed advanced melanoma patients per center

Table S2. Number of newly diagnosed advanced melanoma patients per center and year of diagnosis, excluding uveal and mucosal melanoma

	**Center**	**1**	**2**	**3**	**4**	**5**	**6**	**7**	**8**	**9**	**10**	**11**	**12**	**13**	**14**
**Diagnosis year**	**Total number of patients (N)**	353	225	249	205	452	400	174	165	191	110	142	87	955	112
**2013**	701	68 (19.3)	40 (17.8)	46 (18.5)	34 (16.6)	68 (15.0)	84 (21.0)	30 (17.2)	37 (22.4)	24 (12.6)	21 (19.1)	26 (18.3)	9 (10.3)	192 (20.1)	22 (19.6)
**2014**	728	84 (23.8)	35 (15.6)	50 (20.1)	36 (17.6)	84 (18.6)	79 (19.8)	21 (12.1)	31 (18.8)	40 (20.9)	23 (20.9)	30 (21.1)	12 (13.8)	177 (18.5)	26 (23.2)
**2015**	817	78 (22.1)	52 (23.1)	63 (25.3)	39 (19.0)	100 (22.1)	76 (19.0)	27 (15.5)	37 (22.4)	48 (25.1)	20 (18.2)	33 (23.2)	17 (19.5)	203 (21.3)	24 (21.4)
**2016**	799	63 (17.8)	47 (20.9)	53 (21.3)	44 (21.5)	92 (20.4)	84 (21.0)	49 (28.2)	33 (20.0)	40 (20.9)	22 (20.0)	36 (25.4)	24 (27.6)	181 (19.0)	31 (27.7)
**2017**	775	60 (17.0)	51 (22.7)	37 (14.9)	52 (25.4)	108 (23.9)	77 (19.2)	47 (27.0)	27 (16.4)	39 (20.4)	24 (21.8)	17 (12.0)	25 (28.7)	202 (21.2)	9 (8.0)

Table S3. Multivariable Cox-regression for OS

Table S3. Multivariable Cox-regression model for OS up to 2 years after diagnosis with a frailty of center ID of patients diagnosed between 2013-2017 (model III). Variables with ≥5% missing values are shown as separate categories.

		Multivariable Cox model		
		**HR**	**95%CI**	**P value**
**Age**		1.02	(1.01-1.02)	<0.001
**Gender**	Male	1		
	Female	0.94	(0.87-1.02)	0.098
**ECOG PS**	0-1	1		
	≥2	1.91	(1.71-2.14)	<0.001
	Unknown	1.26	(1.12-1.43)	<0.001
**LDH**	Normal	1		
	250-500 U/L	1.33	(1.20-1.47)	<0.001
	>500 U/L	2.33	(2.06-2.64)	<0.001
	Unknown	0.80	(0.67-0.95)	0.106
**Brain metastases**	No	1		
	Yes. asymptomatic	1.44	(1.26-1.65)	<0.001
	Yes. symptomatic	1.82	(1.65-2.01)	<0.001
**Liver metastases**	No	1		
	Yes	1.31	(1.18-1.44)	<0.001
**Number of organ sites**	0-2	1		
	≥3	1.50	(1.37-1.65)	<0.001
**$BRAF^{V600}$ mutation**	Wild-type	1		
	Mutant	0.85	(0.79-0.93)	<0.001
**New systemic therapy**	No	1		
	Yes	0.66	(0.61-0.72)	<0.001
